# Isolation and pan-genome analysis of *Enterobacter hormaechei* Z129, a ureolytic bacterium, from the rumen of dairy cow

**DOI:** 10.3389/fmicb.2023.1169973

**Published:** 2023-04-06

**Authors:** Huiyue Zhong, Nan Zheng, Jiaqi Wang, Shengguo Zhao

**Affiliations:** State Key Laboratory of Animal Nutrition, Institute of Animal Sciences, Chinese Academy of Agricultural Sciences, Beijing, China

**Keywords:** rumen, ureolytic bacteria, isolation, urea metabolism, pan-genome analysis

## Abstract

**Introduction:**

Urea is an important non-protein nitrogen source for ruminants. In the rumen, ureolytic bacteria play critical roles in urea-nitrogen metabolism, however, a few ureolytic strains have been isolated and genomically sequenced. The purpose of this study was to isolate a novel ureolytic bacterial strain from cattle rumen and characterize its genome and function.

**Methods:**

The ureolytic bacterium was isolated using an anaerobic medium with urea and phenol red as a screening indicator from the rumen fluid of dairy cattle. The genome of isolates was sequenced, assembled, annotated, and comparatively analyzed. The pan-genome analysis was performed using IPGA and the biochemical activity was also analyzed by test kits.

**Results:**

A gram-positive ureolytic strain was isolated. Its genome had a length of 4.52 Mbp and predicted genes of 4223. The 16S rRNA gene and genome GTDB-Tk taxonomic annotation showed that it was a novel strain of *Enterobacter hormaechei*, and it was named *E. hormaechei* Z129. The pan-genome analysis showed that Z129 had the highest identity to *E. hormaechei* ATCC 49162 with a genome average nucleotide identity of 98.69% and possessed 238 unique genes. Strain Z129 was the first *E. hormaechei* strain isolated from the rumen as we know. The functional annotation of the Z129 genome showed genes related to urea metabolism, including urea transport (*urt*A*-urt*E), nickel ion transport (*ure*J, *ton*B, *nix*A, *exb*B, *exb*D, and *rcn*A), urease activation (*ure*A-ureG) and ammonia assimilation (*gdh*A, *gln*A, *gln*B, *gln*E, *gln*L, *gls*A, *glt*B, and *glt*D) were present. Genes involved in carbohydrate metabolism were also present, including starch hydrolysis (*amy*E), cellulose hydrolysis (*cel*B and *bgl*X), xylose transport (*xyl*F*-xyl*H) and glycolysis (*pgi*, *pgk*, *fba*A, *eno*, *pfk*A, *gap*, *pyk*, *gpm*L). Biochemical activity analysis showed that Z129 was positive for alkaline phosphatase, leucine arylamidase, acid phosphatase, naphthol-AS-BI-phosphohydrolase, α-glucosidase, β-glucosidase, and pyrrolidone arylaminase, and had the ability to use D-ribose, L-arabinose, and D-lactose. Urea-nitrogen hydrolysis rate of Z129 reached 55.37% at 48 h of incubation.

**Discussion:**

Therefore, the isolated novel ureolytic strain *E. hormaechei* Z129 had diverse nitrogen and carbon metabolisms, and is a preferred model to study the urea hydrolysis mechanism in the rumen.

## 1. Introduction

The protein level of feed is crucial in ruminant animal growth and production, and the lower economic cost of urea promotes its partial replacement of plant protein in feed (Cherdthong and Wanapat, 2010). Urea recycling is especially important for ruminants because it provides recycled endogenic nitrogen, which is combined with energy for the synthesis of microbial crude protein in the rumen (Reynolds and Kristensen, 2008; Calsamiglia et al., 2010). The microbial crude protein is easily digested, has balanced amino acids (AAs; Schwab and Broderick, 2017), and is also the major component of the metabolizable protein to meet the AA requirement of dairy cows (Owens et al., 2014). The utilization of urea relies on the activity of ureolytic bacteria, but more than 55% of ureolytic bacteria in the rumen are not matched to any known bacterial family (Jin et al., 2016), indicating that many ureolytic bacteria remain to be discovered and isolated. Up to now, 24 strains of rumen ureolytic bacteria have been isolated from various families including Lactobacillaceae, Oscillospiraceae, Staphylococcaceae, Enterobacteriaceae, Bacteroidaceae, and Succinivibrionaceae, Selenomonadaceae (Gibbons and Doetsch, 1958; Slyter et al., 1968; John et al., 1974; Vanwyk and Steyn, 1975; Cook, 1976; Wozny et al., 1977; Lauková and Koniarová, 1995; Cook et al., 2007; Kim et al., 2014; Hailemariam et al., 2020). Among them, only three strains were isolated after 2000 (Cook et al., 2007; Kim et al., 2014; Hailemariam et al., 2020).

Some factors make bacteria isolation and culture difficult, such as symbiosis (St. John et al., 2019), growth factors from other bacteria (D'Onofrio et al., 2010), slow growth (Pulschen et al., 2017), dormancy (Oliver, 2010), competition among strains (Lewis et al., 2020), and media eutrophication (Zhang et al., 2020). There are 1,000 of bacterial species in the rumen (Makkar and Mcsweeney, 2005), but less than 30 ureolytic bacterial species have been isolated (Gibbons and Doetsch, 1958; Slyter et al., 1968; John et al., 1974; Vanwyk and Steyn, 1975; Cook, 1976; Wozny et al., 1977; Lauková and Koniarová, 1995; Cook et al., 2007; Kim et al., 2014; Hailemariam et al., 2020). With the development of sequencing technology, progressively more bacteria have been discovered through culture-free technology and their functional genes have been studied, but the isolation of bacteria is still crucial. Without successful isolation and cultivation of bacteria, the metabolic pathways, physiological characteristics, and ecological functions based on omics data cannot be studied and verified (Gutleben et al., 2018). Therefore, it is crucial to isolate more novel ureolytic bacteria from the rumen to better understand urea metabolism.

In this study, we isolated a new strain of *E. hormaechei* from medium plus urea and named it Z129. We compared Z129 with other strains in the same species and found its unique genes related to nitrogen and carbohydrate metabolisms. We focused on the genes involved in urea metabolism and carbohydrates to explore its ability for urea and carbon metabolisms.

## 2. Materials and methods

### 2.1. Culture media

The liquid media contained 20 ml of clarified rumen fluid, 0.05 g of glucose, 0.05 g yeast extract, 2 g of urea, 0.05 g of cellobiose, 15 mL of solution 4 (0.3% K_2_HPO_4_), 15 mL of solution 5 (0.3% KH_2_PO_4_, 0.6% NaC1, 0.06% MgSO_2_·7H_2_O, and 0.06% CaCl_2_), 0.1 mL of Pfennig trace elements (0.03% H_3_BO_3_, 0.01% ZnSO_4_·7H_2_O, 0.003% MnCl_2_·4H_2_O, 0.002% CoCl_2_·6H_2_O, 0.003% Na_2_MoO_4_·2H_2_O, 0.001% Na_2_SeO_3_, 0.002% NiCl_2_, 0.001% CuCl_2_·2H_2_O, and 0.015% FeCl_2_·4H_2_O), 5 mL of hemin (0.05%), 0.31 mL of VFA mix (17% acetic, 6% propionic, 4% n-butyric, 1% n-valeric, 1% isovaleric, 1% isobutyric, and 1% 2-methyl butyric acids), 0.6 g of NaHCO_3_, 0.1 mL of resazurin (0.1%), 0.05 g of L-cysteine HCl, and 0.0012 g of phenol red per 100 mL. The solid media were the corresponding basal media plus 2 g of agar. After boiling, high-purity nitrogen was blown into the solution for almost 2 h to exhaust the oxygen. After adjusting to pH 6.8, the solution was transferred into an anaerobic workstation (DWS, West Yorkshire, United Kingdom). For liquid media, 10 mL of solution was dispensed into Hungate tubes, and autoclaved at 100 kPa and 121°C lasting for 15 min. After cooling, all media were stored at 4°C. For solid media, agar was added to liquid media and autoclaved at 100 kPa and 121°C lasting for 15 min. After cooling to 40°C–50°C, the solution was poured into 90-mm culture dishes in the anaerobic workstation. Noticeably, after autoclaving, urea and phenol red were added to the media through a 2 μm sterile filter membrane.

### 2.2. Isolation of ureolytic strain

The rumen liquid samples were collected from Holstein dairy cattle (No. IAS2019-14). The inoculum was prepared and stored as described by Hailemariam et al. (2020). The diluent solution contained 15 mL of solution 4, 15 mL of solution 5, 0.6 g of NaHCO_3_, 0.1 mL of resazurin (0.1%), and 0.05 g of L-cysteine HCl per 100 mL, and was prepared under anaerobic conditions in the anaerobic workstation. The inoculum was diluted 10 to 10^5^ times with the anaerobic diluent. Then 200 μL of diluted inoculum in each dilution was spread on the solid media. After incubating for 72 h at 39°C, colonies with pink color were selected and streaked in new solid media. The single pink colonies were inoculated into liquid media. The strain was mixed with equal anaerobic diluent solution containing 30% glycerin, and stored at −80°C.

### 2.3. 16S rRNA gene sequencing

The DNA of isolated strains was extracted by cetyltrimethylammonium bromide (CTAB) plus bead beating method as described by Minas et al. (2011). The primers were 27F (5′-AGAGTTTGATCMTGGCTCA-3′) and 1492R (5′-TACGGYTTACCTTGTTACGACTT-3′). The PCR mixture included 25 μL of PCR Master Mix (Takara, Japan), 0.1 μL of 27F, 0.1 μL of 1492R, 5 μL of microbial DNA, and 19.8 μL of ddH_2_O. The process of amplification was same as the described by Hailemariam et al. (2020). The PCR products were sequenced by Sanger sequencing (Applied Biosystems 3730XL, Foster City, CA, United States). The chromas were used to determine whether the strain was pure or contaminated according to single or double peak on the position of each base. Only pure strains were selected for genome sequencing and biochemical analysis. The 16S rRNA sequences were blasted with the rRNA/ITS database of NCBI for taxonomic annotation.

### 2.4. Physiological characterization

The test for Gram-staining was performed using a kit, following the manufacturer’s instructions (RealTime Biotechnology, Beijing, China). The Gram-staining result was observed using a bright-field microscope (Axiocam ERc 5 s, ZEISS, Oberkochen, Germany).

The enzyme activity and the ability for sugar fermentation were tested by API ZYM (Merieux, Lyon, France) and API 20 STREP (Merieux, Lyon, France). The inoculum samples were taken at 0 and 48 h of incubation to measure the content of urea nitrogen using a BUN test kit with the diacetyloxime colorimetric method (Jiancheng, Nanjing, China).

### 2.5. Whole genome sequencing and analysis

The DNA of Z129 was extracted as the above and quantified using Qubit 2.0 (Invitrogen, Carlsbad, CA, United States). The sequencing library was constructed using a MGIEasy Universal DNA library kit (BGI, Shenzhen, China). The libraries were paired-end (2 × 150 bp) sequenced by DNBseq-T7 (BGI). The low quality reads including reads with length lower than 50 bp and reads with average mass lower than 20 were removed by TrimGalore (Martin, 2011). The qualified data were assembled to produce genomes using Megahit (Li et al., 2015), then the quality and contamination were checked using checkM (Parks et al., 2015). The taxonomy was annotated by GTDB-tk (Chaumeil et al., 2020) based on the GTDB RS202 database. The genes were predicted using Prokka (Seemann, 2014). The proteins were aligned to the eggNOG 5.0 database using diamond (Buchfink et al., 2014). The genes related to urea nitrogen and carbohydrate metabolisms were identified. CAZymes annotation was performed on the dbCAN meta server (Yin et al., 2012).

### 2.6. Pan-genome analysis

Based on the taxonomic result of whole genome sequencing, the genome sequences of 12 strains (*E. hormaechei* YT3, *E. hormaechei* YT2, *E. hormaeche*i subsp. *hoffmannii* UCICRE 9, *E. hormaechei* subsp. *hoffmannii* UCICRE 3, *E. hormaechei* subsp. *hoffmannii* UCI 50, *E. hormaechei* subsp. *hoffmannii* MGH 13, *E. hormaechei* subsp. *hoffmannii* ECNIH3, *E. hormaechei* subsp. *hoffmannii* ECR091, *E. hormaechei* subsp. *steigerwaltii*, *E. hormaechei* subsp. *xiangfangensis*, *E. hormaechei* ATCC 49162, *E. hormaechei* subsp. *oharae*) on NCBII database were downloaded. Pan-genome analysis was performed using IPGA (Liu et al., 2022). The AA sequences of UreC of these strains were also downloaded from the NCBI database and aligned using the CLUSTAL W package in BioEdit (Alzohairy, 2011).

## 3. Results

### 3.1. Isolation and genome of ureolytic strain

**Figure 1:**
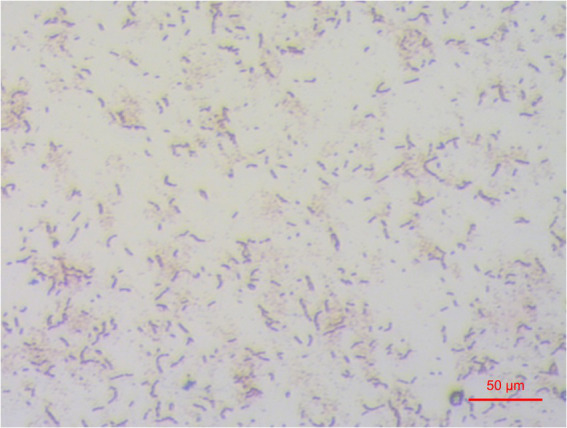
Gram-staining of strain Z129 observed by a bright-field microscope.

**Figure 2:**
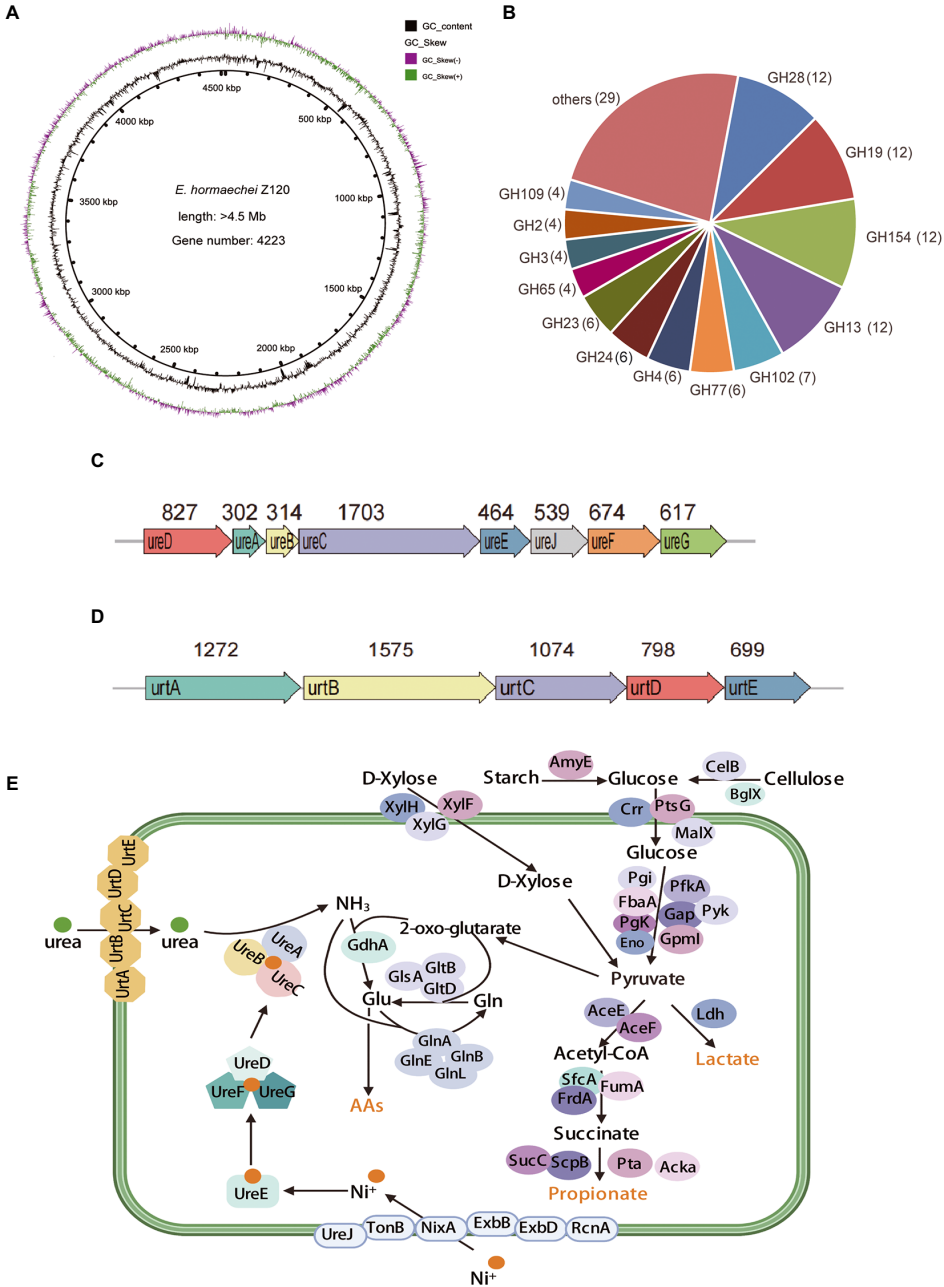
Functional genes related to urea and carbohydrate metabolism of strain Z129. **(A)** Circle diagram showing the genome. From the outside to the inside are GC-skew and GC-content. **(B)** The number of annotated genes in different GH families. The GH families with less than four genes were combined into the others. **(C)** Gene clusters for urea hydrolysis. The number indicates the length of the gene, the unit is bp. **(D)** Gene clusters for urea transportation. The number indicates the length of the gene, the unit is bp. **(E)** Genes involved in urea transport, nickel ions transport, urea hydrolysis, ammonia assimilation, starch metabolism, cellulose metabolism, xylose metabolism, and pyruvate metabolism.

According to the color indicator and 16S rRNA gene sequence chromas, one isolate Z129 with ureolytic activity was finally obtained, and was identified as a gram-positive bacterium (Figure 1). The genome of the isolate had a size of 4.52 Mbp, with completeness of 99.97% and contamination of 0.33% assessed by checkM (Figure 2A). There were 177 contigs with maximum length of 426 kb, minimum of 201 bp, and average of 25.5 kb. The number of predicted genes was 4,223, of which 4,074 were annotated using eggNOG database (Figure 2A). The GTDB-tk taxonomic analysis showed that Z129 belonged to *E. hormaechei*. The 16S rRNA gene showed that Z129 was most closely related to *E. hormaechei* subsp. *xiangfangensis* with identity of 99.36%.

### 3.2. Genes involved in urea and carbohydrate metabolism

A total of 30 GH families involved in carbohydrate metabolism were identified in the genome of Z129. The predominant members were GH13, GH19, GH28, and GH154 and account for 40% of the total (Figure 2B). The genes involved in urea hydrolysis including *ure*A (302 bp), *ure*B (314 bp), *ure*C (1,703 bp), *ure*D (827 bp), *ure*E (464 bp), *ure*F (674 bp), and *ure*G (617 bp) and the gene *ure*J (539 bp) involved in nickel ion transport were clustered together (Figure 2C). Additionally, genes involved in urea transport were also clustered together: *urt*A-*urt*E (Figure 2D). Urea metabolism related genes in the Z129 genome were grouped as genes involved in urea transport (*urt*A-*ure*E), nickel ions transport (*ure*J, *ton*B, *nix*A, *exb*B, *exb*D, and *rcn*A), urea hydrolysis (*ure*A, *−ure*G, and *ure*J), ammonia assimilation (*gdh*A, *gln*A, *gln*B, *gln*E, *gln*L, *gls*A, *glt*B, and *glt*D; Figure 2E). Carbohydrate metabolism related genes were grouped as genes related to starch hydrolysis (*amy*E), cellulose hydrolysis (*cel*B and *bgl*X), glucose transport (*crr*, *prs*G, and *mal*X), xylose transport (*xyl*F-*xyl*H), glycolysis (*pgi*, *pgk*, *fba*A, *eno*, *pfk*A, *gap*, *pyk*, and *gpm*L), and short-chain fatty acid biosynthesis (*ace*E, *ace*F, *frd*A, *sfc*A, *suc*C, *scp*B, *acka*, and *pta*; Figure 2E). As for the biosynthesis of amino acids such as asparagine, cysteine, methionine, lysine, alanine, and threonine, genes including *asn*B, *san*A, *cysk1*, *yxj*G, *dapf*, *isc*S, and *thr*C were also identified in the Z129 genome.

### 3.3. Pan-genome analysis of *Enterobacter hormaechei*

**Figure 3:**
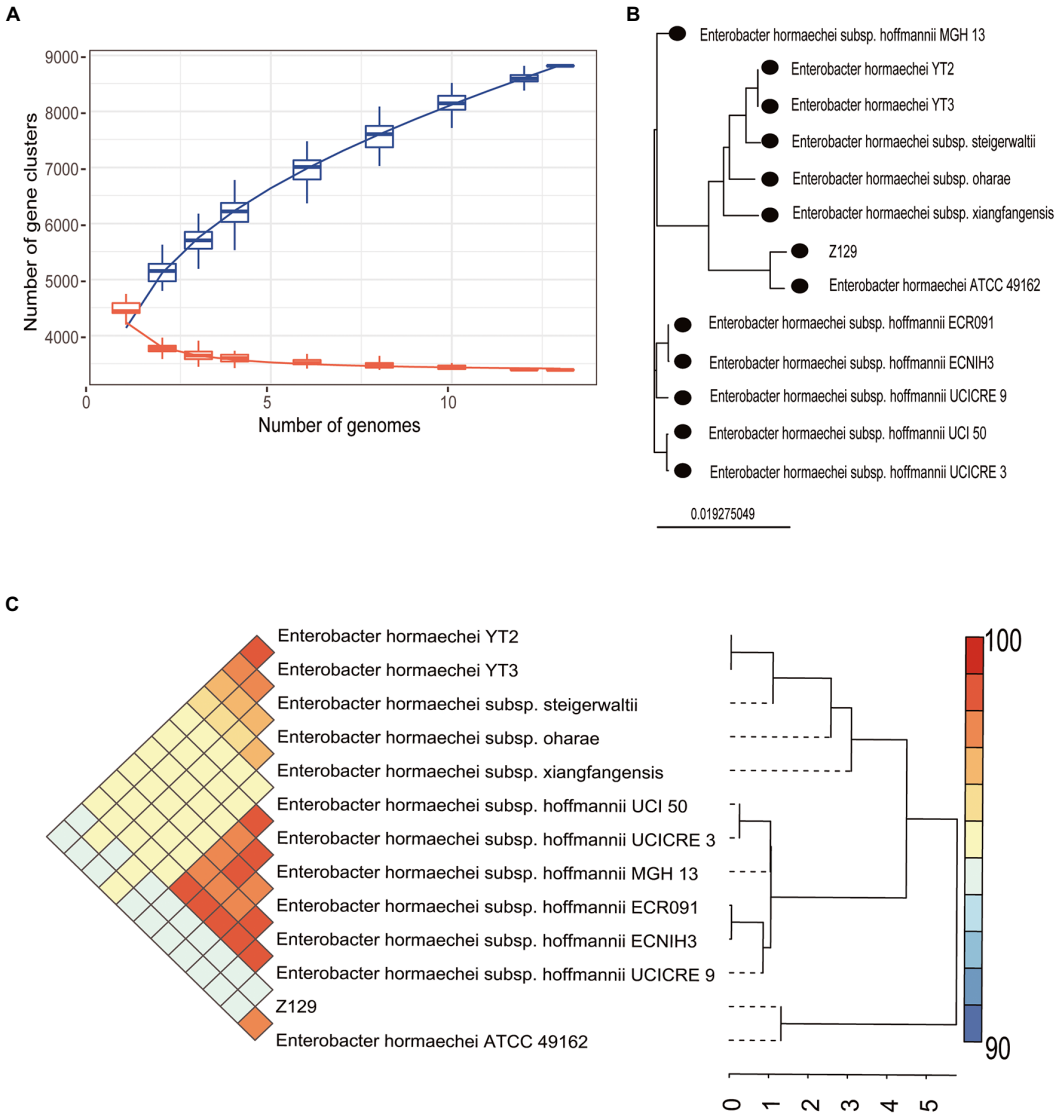
Pan-genome analysis of Z129 and 12 other *Enterobacter hormaechei* strains. **(A)** The number of pan gene clusters and core gene clusters along with the addition of new strains of *E. hormaechei*. **(B)** Phylogenetic tree highlighting the relationship of Z129 with other *E. hormaechei* strains based on the genome sequence. **(C)** Pairwise comparisons of average nucleotide identity (ANI). The color indicates the value of ANI, the value range is 90–100 with color turning from blue to red.

**Figure 4:**
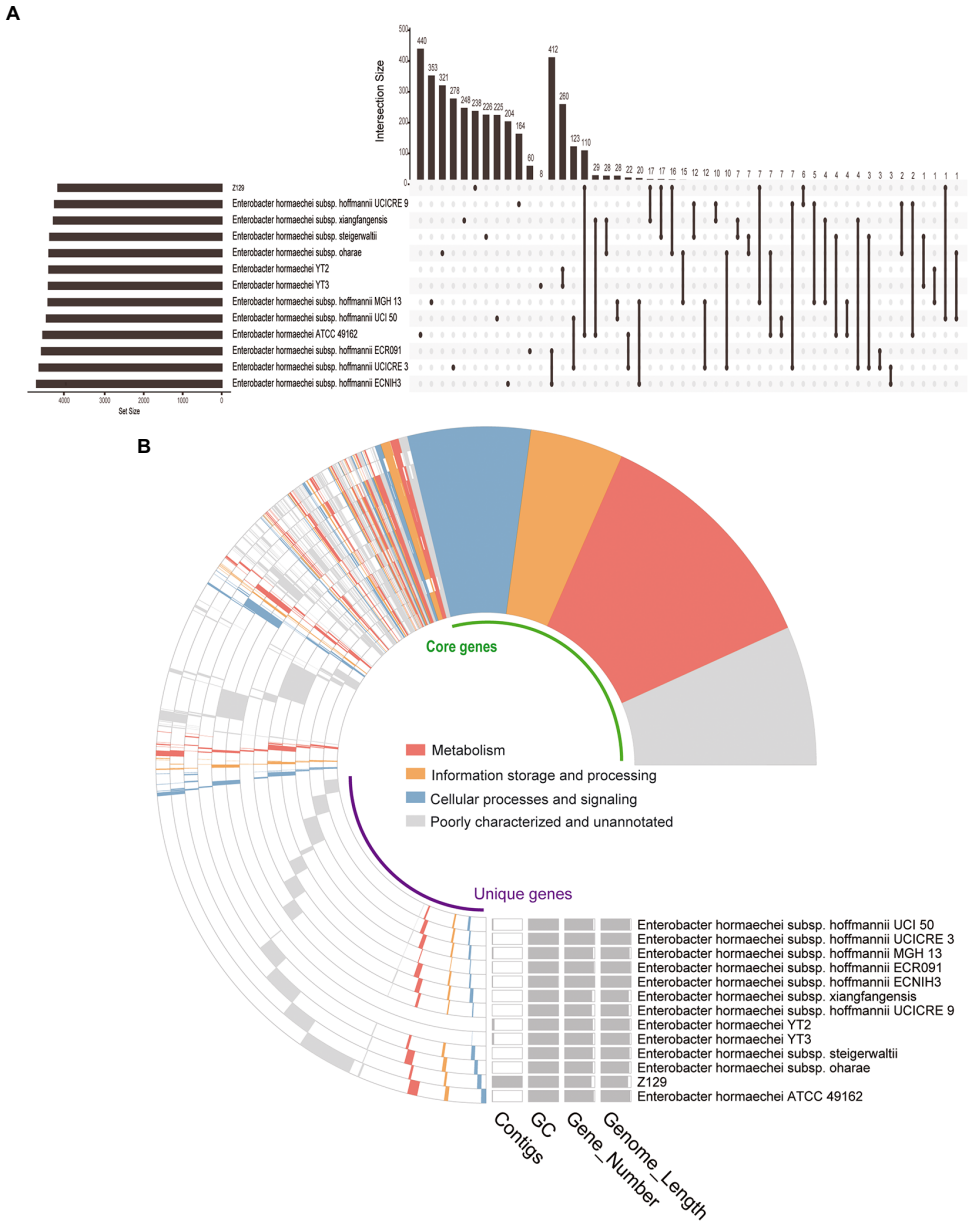
Distribution of genes among *Enterobacter hormaechei* strains. **(A)** Upset figure showing the unique genes of each strain and genes shared between any two strains. **(B)** COG annotation showing the core genes, unique genes, number of contigs, GC content, and genome length.

**Figure 5:**
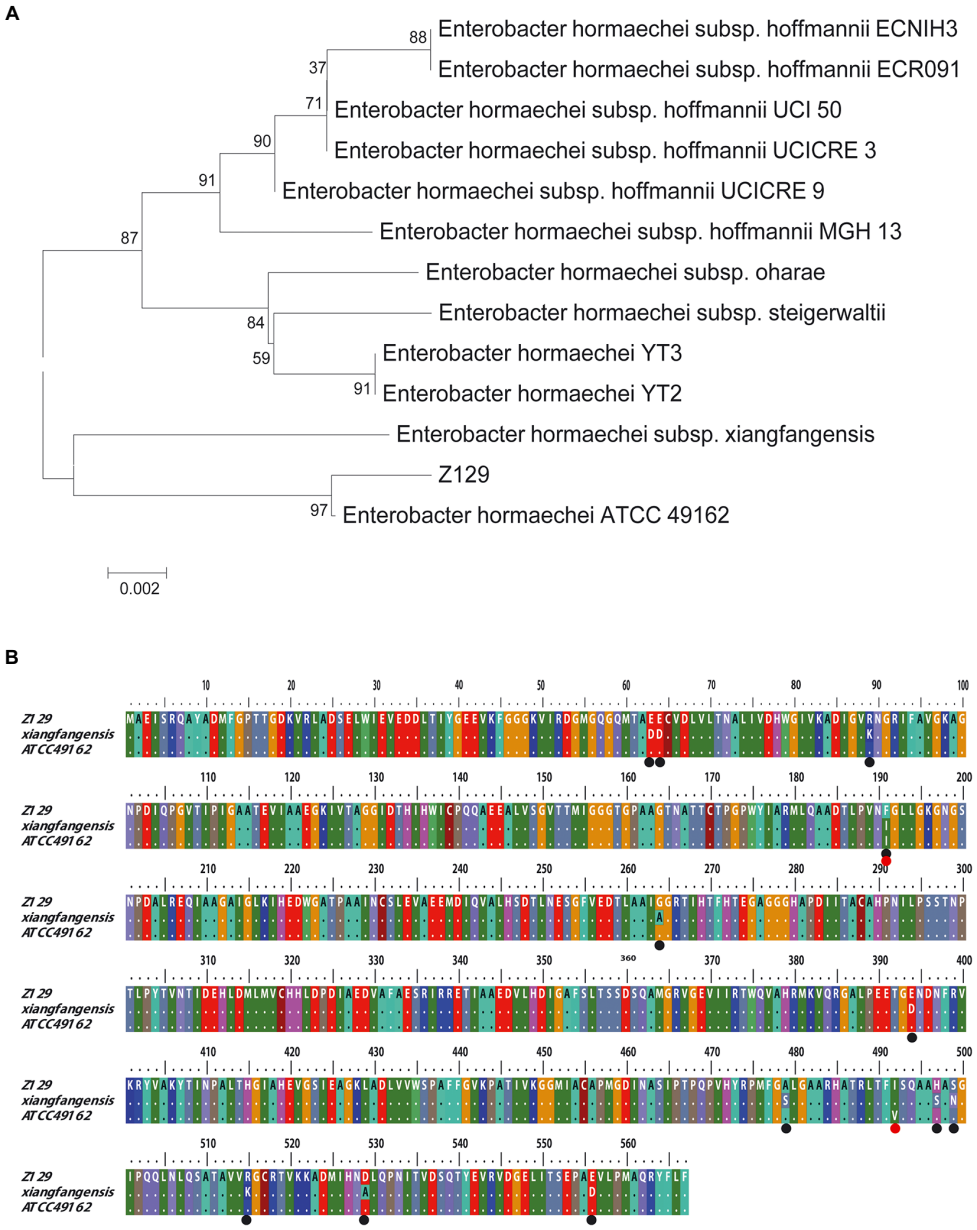
Comparative analysis of Z129 and 12 other *Enterobacter hormaechei* strains based on UreC AA sequence. **(A)** Phylogenetic tree based on UreC AA sequence. **(B)** Alignment of UreC AA sequences among Z129, *E. hormaechei* subsp. *xiangfangensis* and *E. hormaechei* ATCC 49162. The black circles indicate the different sites between Z129 and *E. hormaechei* subsp. *xiangfangensis*, and the red circles indicate the different sites between Z129 and *E. hormaechei* ATCC 49162.

Twelve genomes of known *E. hormaechei* strains and Z129 were used for pan-genome analysis. The number of pan gene clusters increased to 8,814 and core gene clusters decreased to 3,388, but the curve gradually flattened out (Figure 3A). The genomic phylogenetic tree analysis of *E. hormaechei* strains also showed that strain Z129 was most closely related to *E. hormaechei* ATCC 49162 (Figure 3B). The average nucleotide identity (ANI)values between Z129 and other strains are as following: *E. hormaechei* YT3 (95.00%), *E. hormaechei* YT2 (94.83%), *E. hormaeche*i subsp. *hoffmannii* UCICRE 9 (94.34%), *E. hormaechei* subsp. *hoffmannii* UCICRE 3 (94.24%), *E. hormaechei* subsp. *hoffmannii* UCI 50 (94.27%), *E. hormaechei* subsp. *hoffmannii* MGH 13 (94.25%), *E. hormaechei* subsp. *hoffmannii* ECNIH3 (94.28%), *E. hormaechei* subsp. *hoffmannii* ECR091 (94.25%), *E. hormaechei* subsp. *steigerwaltii* (94.80%), *E. hormaechei* subsp. *xiangfangensis* (94.75%), *E. hormaechei* ATCC 49162 (98.69%), *E. hormaechei* subsp. *oharae* (95.06%; Figure 3C). The ANI value between Z129 and *E. hormaechei* ATCC 49162 was the highest. The pan-genome analysis among 13 genomes showed that the amount of unique genes in each genome was in the range of 8–440 (Figure 4A). Strain Z129 provided 238 new genes representing 2.7% of genes of the species, and 5.6% of genes of its genome (Figure 4A). Compared with the other 12 strains, Z129 shared 110 genes with the most closely related strain *E. hormaechei* ATCC 49162 and shared no genes with *E. hormaechei* YT3, *E. hormaechei* YT2, *E. hormaechei* subsp. *hoffmannii* ECR091, UCICRE 3, and ECNIH3 (Figure 4A). The COG annotation showed that the core gene clusters included 1,438 genes for metabolism, 767 for cellular processes and signaling, 573 for information storage and processing and 610 for not annotated and poorly characterized (Figure 4B). The shared genes between Z129 and *E. hormaechei* ATCC 49162 included 19 genes for metabolism, 6 for cellular processes and signaling, 31 for information storage and processing and 54 for not annotated and poorly characterized (Figure 4B). Some of unique genes, such as *appA*, *nrfa*, and *wecE*, of Z129 were related to phosphorus and nitrogen metabolisms (Supplementary Table 1). Additionally, urease protein UreC existed in all *E. hormaechei* strains and the phylogenetic tree of AA sequences showed that Z129 was most closely related to *E. hormaechei* ATCC 49162 followed by *E. hormaechei* subsp. *xiangfangensis* (Figure 5A). Two sites in the UreC AA sequence differed between Z129 and *E. hormaechei* (Figure 5B).

### 3.4. Functional activity of Z129

**Figure 6:**
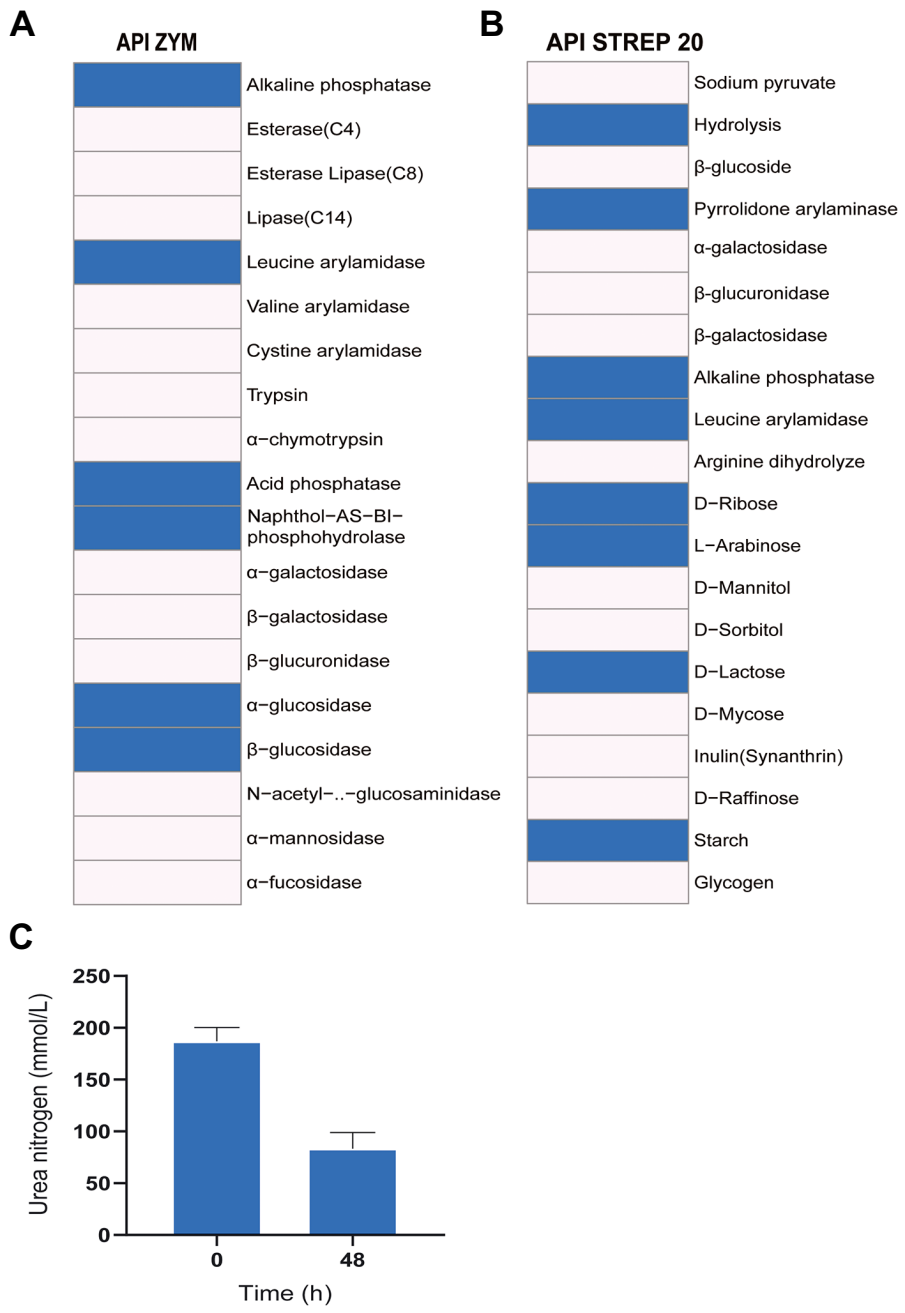
The enzyme activity and utilization of carbohydrates and urea. **(A)** Results of API ZYM, indicating 19 kinds of enzyme activities. The blue presents positive, the blank presents negative. **(B)** The results of API STREP 20. The result indicates enzyme activities and utilization of carbohydrates. The blue presents positive, the blank presents negative. **(C)** Change of urea nitrogen with the increased culturing time.

The enzyme activity test using API ZYM and API 20 STREP showed that Z129 was positive for alkaline phosphatase, leucine arylamidase, acid phosphatase, naphthol-AS-BI-phosphohydrolase, α-glucosidase, β-glucosidase, and pyrrolidone arylaminase, but negative for esterase (C4), esterase lipase (C8), lipase (C14), valine arylamidase, cystine arylamidase, trypsin, α-chymotrypsin, α-galactosidase, β-galactosidase, β-glucuronidase, N-acetyl-β-glucosaminidase, α-mannosidase, and α-fucosidase (Figures 6A, B). The sugar utilization assay of API 20 STREP revealed that Z129 took advantage of D-ribose, L-arabinose, D-lactose, and starch, but not D-mannitol, D-sorbitol, D-trehalose, inulin, D-raffinose, and glycogen. The urea nitrogen during Z129 incubation was decreased by 55.37% at 48 h indicating high ureolytic activity (Figure 6C).

## 4. Discussion

We isolated a new strain Z129 belonging to *E. hormaechei* that showed an ANI of 98.69% with *E. hormaechei* ATCC 49162. Compared with the strains of this species, Z129 possessed unique genes including *app*A, *nrf*A, and *wec*E. The expression of *app*A significantly improves phytase activity (Chiera et al., 2004), which can increase the efficiency of phytate phosphorus (Beaulieu et al., 2007). The addition of phytases improves the final body weight and feed conversion ratio of weaned piglets fed with a diet lacking in calcium and phosphorus (Wiśniewska et al., 2020). Gene *nrf*A can be involved in catalysis of the second step of dissimilatory nitrate reduction to ammonium to reduce nitrogen loss and protects the environment (Tiedje, 1988). Expression of *nrf*A is induced in a low-nitrate environment (Wang and Gunsalus, 2000). Gene *wec*E has been identified in *Escherichia coli* and encodes a sugar aminotransferase regulating synthesis of TDP-4-amino-4,6-dideoxy-D-galactose (Hwang et al., 2004). Therefore, the isolation of Z129 enriches taxonomic information, and the discovery of these unique genes expands the known genetic diversity within *E. hormaechei*.

Strains of *E. hormaechei* can produce urease (Roslan et al., 2020) and all *E. hormaechei* strains possesses genes encoding urease. Our results also showed that Z129 could utilize urea nitrogen. However, there is limited research concerning the genes involved in the whole urea metabolism within *E. hormaechei*. In this study, we successfully explored these genes. Urease is crucial in urea metabolism. Although urease gene clusters differ for different bacteria, they usually include structural genes (e.g., *ure*A-*ure*C) and accessory genes (e.g., *ure*D-*ure*I). Urease is a metalloenzyme with two nickel ions in its active center (Alfano and Cavazza, 2020). In fact, urease activation is a metal assembly process between nickel ions and urease structural proteins. Firstly, UreE transfers nickel ions to UreG (Yang et al., 2015), then UreG passes nickel ions to UreF (Zambelli et al., 2014), and nickel ions finally enter urease through UreD channels to complete the assembly of metal centers (Carter and Hausinger, 2010). Therefore, these helper genes and nickel ions are essential for urea metabolism. The entry of nickel ions is aided by nickel ion transporters encoded by *nik*A-*nik*E, *fec*ADE, *frpB4*, *ton*B, *Exb*BD, *nix*A, *ceu*E, *hpn*, and *ure*J (Dmitry et al., 2006; Hilde et al., 2013; Haley and Gaddy, 2015; Vinella et al., 2015). In this study, genes related to urease (*ure*A-*ure*G) and related to nickel ion transport (*ure*J, *ton*B, *nix*A, *exb*B, *exb*D, and r*cn*A) were all detected. The urease of rumen bacteria is located in the cytoplasm (McLean et al., 1985), meaning that urea must enter the bacteria before it is hydrolyzed by urease. Rumen bacteria usually possess three types of urea transporters: one is pH-independent, such as Yut (Sebbane et al., 2002), the other is ATP-activated encoded by *urt*A-*urt*E (Hailemariam et al., 2021). In strain Z129, we identified genes including *urt*A-*urt*E related to urea transport. The urea is hydrolyzed to ammonia which is not used directly by ruminants. The ammonia should be assimilated to produce other forms of nitrogen for the growth and production of ruminants. The nitrogen assimilation in bacteria is controlled by *gdh*A, *gln*A, *gln*N, *gln*B, *gln*E, *gln*G, *gln*L, *gln*K, *gln*R, and *gls*A (Herrero et al., 2019; Liu et al., 2020), which are also found in Z129 genome. All these results revealed that strain Z129 contained all the genes involved in urea metabolism including urease activation, urea transport, and ammonia assimilation, which meant that Z129 possessed the ability for urea metabolism. The rumen fermentation *in vitro* also showed that Z129 had ureolytic activity.

In addition to the urea metabolism, we also focused on the carbohydrate utilization of Z129. The *E. hormaechei* species can ferment various carbohydrates including D-glucose, L-arabinose, cellobiose, dulcitol, D-galactose, maltose, D-mannitol, D-mannose, L-rhamnose, sucrose, trehalose, and D-xylose (O'Hara et al., 1989). The sugar utilization test also showed that Z129 fermented D-ribose, L-arabinose, and D-lactose but not D-mannitol, D-sorbitol. D-trehalose, inulin, D-raffinose, and glycogen. Although there were some differences from the result of O'Hara et al. (1989), Z129 showed the ability to ferment various carbohydrates. The CAZymes annotation also showed that there were various GH families including GH13, GH19, GH154, GH28, GH102, GH77, GH4, GH24, and GH23 in the Z129 genome. The GH28 family plays important roles in pectin degradation (Zhao et al., 2013) and contains all pectin-degrading hydrolases (Anuradha and Bhawaniprasad, 2019). The GH19 family contains endo-chitinases which hydrolyzes the chitinoside bond to produce N-acetyl-D-glucosamine (Nakagawa et al., 2013). The most typical and studied enzyme among GH13 is α-amylase, which specifically catalyzes hydrolysis of α-1,4-glycosidic linkages of starch to produce small molecular products including glucose, maltose, and maltotriose (Gangadharan et al., 2008). In addition, genes related to starch, cellulose and hemicellulose metabolism including *amy*E, *cel*B, *bgl*X, *xyl*F-*xyl*H, *fba*A, *ace*A, and *suc*C were also identified in the Z129 genome. The nitrogen from urea metabolism and the carbon from carbohydrate fermentation are used together to produce various AAs.

In conclusion, Z129 is a new gram-positive strain of *E. hormaechei*, carrying unique genes including *app*A, *nrf*A, and *wec*E related to feed conversion ratio, nitrogen dissimilatory reduction and lactose synthesis. Strain Z129 possesses the genes of each step of urea metabolism and various genes involved in cellulose, hemicellulose, and starch fermentation.

## Data availability statement

The names of the repository/repositories and accession number(s) can be found at: https://nmdc.cn/en, SUB1675681571147 and https://www.ncbi.nlm.nih.gov/, GCF_001875655.1, GCF_000750225.1, GCF_000750275.1, GCF_000534575.1, GCF_000534035.1, GCF_000492615.1, GCF_000492495.1, GCF_020097195.1, GCF_001729725.1, GCF_001729785.1, GCF_000328905.1, and GCF_000328885.1.

## Ethics statement

The animal study was reviewed and approved by the Ethics Committee of Institute of Animal Sciences of CAAS. Written informed consent was obtained from the owners for the participation of their animals in this study.

## Author contributions

SZ designed the study and reviewed the paper. HZ performed the experiments and wrote the paper. SZ and HZ analyzed the genome equally. JW and NZ contributed to project administration and funding acquisition. All authors contributed to the article and approved the submitted version.

## Funding

National Natural Science Foundation of China (32272888), the Agricultural Science and Technology Innovation Program (ASTIP-IAS12), and State Key Laboratory of Animal Nutrition (2004DA125184G2108).

## Conflict of interest

The authors declare that the research was conducted in the absence of any commercial or financial relationships that could be construed as a potential conflict of interest.

## Publisher’s note

All claims expressed in this article are solely those of the authors and do not necessarily represent those of their affiliated organizations, or those of the publisher, the editors and the reviewers. Any product that may be evaluated in this article, or claim that may be made by its manufacturer, is not guaranteed or endorsed by the publisher.
